# Botulism Cases in Romania—An Overview of 14-Year National Surveillance Data

**DOI:** 10.3390/biomedicines12051058

**Published:** 2024-05-10

**Authors:** Andreea Marilena Păuna, Maria-Dorina Crăciun, Anca Sîrbu, Rodica Popescu, Bianca Georgiana Enciu, Carmen-Daniela Chivu, Mădălina Simoiu, Daniela Piţigoi

**Affiliations:** 1 Department of Epidemiology I, Carol Davila University of Medicine and Pharmacy, 020021 Bucharest, Romaniamaria.craciun@umfcd.ro (M.-D.C.); daniela.pitigoi@umfcd.ro (D.P.); 2 Military Medical Institute, 010919 Bucharest, Romania; 3 Emergency Clinical Hospital for Children “Grigore Alexandrescu”, 011743 Bucharest, Romania; 4 Department of Epidemiology II, Carol Davila University of Medicine and Pharmacy, 020021 Bucharest, Romaniabianca.milcu@drd.umfcd.ro (B.G.E.); 5 National Centre for Communicable Diseases Surveillance and Control, National Institute of Public Health, 050463 Bucharest, Romania; 6 Department of Parasitology, Carol Davila University of Medicine and Pharmacy, 020021 Bucharest, Romania; 7 National Institute for Infectious Diseases “Prof. Dr. Matei Balș”, 021105 Bucharest, Romania

**Keywords:** botulism cases, surveillance, Romania

## Abstract

Botulism is a priority disease worldwide because it has a very severe course of evolution that can lead to death. This paper aims to describe the main epidemiological characteristics of botulism cases confirmed in Romania over 14 years (2007–2020). We performed a retrospective study using the publicly available national surveillance data and reported to the National Institute of Public Health. A total of 325 cases of foodborne botulism were reported in Romania, with no infant or wound botulism. Most of the cases (125, 38.5%) were reported among young adults (25–44 years old), over half (205, 63%) of them living in rural areas. The incriminated food item was identified in 161 cases; in most cases (145, 90%) the food item was prepared in the household. The main food category was represented by meat and meat-based products (94, 68.6%). In almost all cases the identified type was BoNT/B (230/231, 99.5%). Fifteen deaths were recorded, and the case fatality rate was 4.6%. Botulism cases were reported annually in Romania. Surveillance data are essential for implementing control measures and adapting educational campaigns according to existing needs.

## 1. Introduction

Botulism is a priority disease worldwide as it has a very severe course of evolution that can lead to the death of the patient in the absence of early diagnosis and prompt treatment. It is not a commonly encountered disease and it is caused by the botulinum toxin (BoNT) produced by *Clostridium botulinum* (*C. botulinum*) and, less commonly, by *Clostridium baratii* (*C. baratii*) or *Clostridium butyricum* (*C. butyricum*) [1]. The *C. botulinum* spores, which are metabolically latent, are widespread in the environment and are highly resistant to various environmental conditions, allowing their persistence in unfavorable conditions. Under anaerobiosis conditions, the spores can germinate and subsequently release the neurotoxin (BoNT) [2,3]. Regarding their antigenicity, there are seven known types of BoNT (designated from A to G) and several subtypes. Toxin subtypes A, B, E, and F cause botulism in humans as well as in animals, while BoNT/B and E cause disease in domestic animals [4,5]. Human botulism cases are mainly determined by BoNT types A and B, rarely E and occasionally F. On the other hand, BoNT types that cause animal botulism are C, D, and their mosaic variants C/D and D/C. In domestic and wild bird species, all these types of toxins have been identified, including in outbreaks, but the C/D mosaic form is more prevalent. BoNT D/C is the predominant type in cattle and is currently the main cause of bovine botulism cases in Europe [6,7]. A new toxin type H (BoNT/H), produced by a *C. botulinum* strain isolated from a case of infant botulism, was discovered in the United States in 2013 [8].

The action of the toxin takes place at the presynaptic level, blocking the release of acetylcholine at the neuromuscular junction level [1,2]. Thus, the clinical picture of the disease includes symmetrical descending flaccid paralysis with a risk of respiratory failure, while in some particularly severe cases it can result in the death of the patient [3,5]. Six clinical forms of the disease have been described, with the most important being foodborne botulism. This clinical form occurs following the ingestion of food products containing the pre-formed toxin, such as those poorly preserved, which have been canned and preserved at home or with incorrect sterilization techniques [9,10]. Another clinical form of the disease is intestinal botulism which occurs following the ingestion of *C. botulinum* spores. Later, the bacterium changes into a vegetative form and releases the toxin at the intestinal level. This form can occur in adults (adult intestinal colonization) or in children under 1 year of age (infant botulism) [9,11]. Wound botulism is produced by the contamination of wounds with *C. botulinum* spores, which later change into a vegetative form capable of secreting toxins. In recent years, cases of wound botulism have been described in intravenous drug users [10,12]. There are two other clinical forms of the disease that do not occur under natural conditions. Inhalation botulism results from accidental or deliberate inhalation of aerosolized botulinum neurotoxin (e.g., in cases of bioterrorism). In the context of the widespread use of botulinum toxin for therapeutic or cosmetic purposes, cases of iatrogenic botulism have been reported in recent years [2,13]. Although the diagnosis is mainly clinical, the performance of laboratory tests with the identification of the BoNT from biological and food samples is mandatory to confirm suspected cases [14].

The treatment of botulism is challenging, and the neutralization of circulating BoNT is essential. Therefore, an early diagnosis and a rapid administration of the antitoxin increase the chances of therapeutic success. Cases with severe evolution may require intensive care, intubation, and mechanical ventilation [11,15].

In general, botulism occurs sporadically, but outbreaks of foodborne botulism are periodically reported, mostly associated with the consumption of home-made food items. However, there were also reported outbreaks of foodborne botulism with international implications [16,17].

Because of its high potential impact on public health, the evolution of the disease is carefully monitored [1]. Additionally, the toxin produced by *C. botulinum* requires permanent attention from the international community, including monitoring through civil–military cooperation (CIMIC). This toxin is the most powerful biological toxin and the biological agent of choice used for armed or terrorist purposes [2,18,19,20].

According to the Annual Epidemiological Report for 2021 of the European Center for Disease Prevention and Control (ECDC), botulism is rarely reported in the European Union but continues to occur sporadically or in small outbreaks, with various clinical forms [2].

In Romania, botulism is included in the list of priority diseases for the surveillance system and is subject to the early warning and rapid reaction system, with an alert threshold of one case [21].

According to a government decision, after specifying the diagnosis of a probable or confirmed case of botulism, it is mandatory that the physician who discovered the case (the general practitioner or any physician in healthcare units, including the laboratory that investigated the samples, those from the social assistance units) report immediately (by telephone) any case of botulism to the surveillance and control service of communicable diseases within the county’s public health department. To implement the reporting system, physicians are required to fill in two forms: the Communicable Disease Case Reporting Form which contains minimal data regarding the suspected case, and a specific surveillance form based on the methodology for the surveillance of foodborne diseases developed by the National Institute of Public Health, which provides information regarding the demographic characteristics of sporadic or multiple cases from clusters and laboratory results in the case of foodborne botulism [21,22]. For every suspected case of botulism, an epidemiological investigation is carried out.

The aim of our study is to describe the epidemiological characteristics of botulism in Romania for a period of 14 years between 2007 and 2020, based on surveillance data.

## 2. Materials and Methods

We performed a descriptive retrospective analysis of botulism cases reported in Romania between 2007 and 2020. We included in the analysis all reported cases (confirmed and probable) in this study period.

### 2.1. Botulism Surveillance System in Romania: Case Definition

In Romania, the case definition used in the surveillance of botulism is in accordance with the Decision EU 945 of 22 June 2018 [23]. The clinical criteria are considered complete for foodborne and wound botulism if there is at least one of the following two: bilateral cranial nerve impairment (diplopia, blurred vision, dysphagia, bulbar weakness) or peripheral symmetric paralysis. Infant botulism should meet at least one of the following six clinical symptoms: constipation, lethargy, difficulty in sucking or feeding, ptosis, dysphagia, and general muscle weakness. The laboratory criteria include at least one of the following three: the isolation of BoNT-producing clostridia (for example, *C. botulinum*, *C. baratii*, *C. butyricum*) for infant botulism (stool) or wound botulism (wound), the detection of botulinum neurotoxins in a clinical specimen, and the detection of genes encoding for botulinum neurotoxins in a clinical specimen. The epidemiological criteria require at least one of the following two epidemiological links to be established: exposure to a common source (for example, food, sharing of needles or other devices), and exposure to contaminated food or drinking water. Cases are classified as probable cases if the clinical criteria and epidemiological link are met and confirmed cases if the clinical and laboratory criteria are met.

### 2.2. Data Collection and Analysis

Data were collected from annual reports of the National Center for the Surveillance and Control of Communicable Diseases (CNSCBT), between 2007 and 2020 [2,15,24]. To complete the characterization of the cases, some data not included in these annual reports were made available by the National Institute of Public Health. These data were processed following the General Data Protection Regulation, with no confidential information regarding cases being shared [25].

We performed a descriptive analysis using the demographic and epidemiological data related to botulism cases. Data regarding the annual number of cases (probable and confirmed—total, by age group, by gender, by month), the annual incidence rate, the annual number of deaths as well as the identified type of toxin were collected from the annual reports. Data regarding the incriminated food items were provided on request. The parameters analyzed in the study were the following: the number of confirmed cases per year, the annual incidence, the number of confirmed cases per month, the number of confirmed cases by counties, the distribution of cases by age groups, the distribution of cases according to the incriminated food item, the number of deaths per year, the distribution of deaths according to gender, and the distribution of deaths according to the residence. Data collection, analysis, and visualization were performed using Microsoft Excel 365, Version 2108.

## 3. Results

### 3.1. Number of Botulism Cases and Annual Incidence Rate

In the study period, 511 cases were reported as suspected cases. Out of them, 325 (63.6%) were classified as either probable cases or confirmed cases. There were 289 (88.9%) confirmed cases and 36 (11.1%) probable cases, all of foodborne botulism.

During 2015–2020, there were 6 familial outbreaks with a total number of 13 cases.

**Figure 1:**
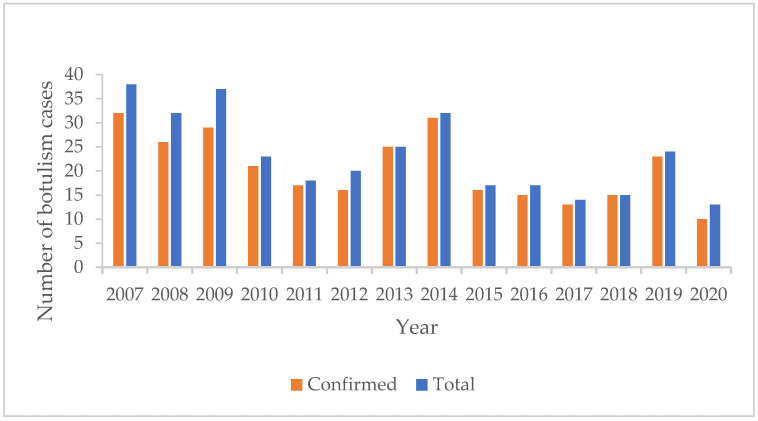
Distribution of botulism cases by year (*n* = 325) in Romania, 2007–2020.

The median number of cases reported was 21.5 cases per year, with an interquartile range (IQR) of 17–32. The maximum number of cases (38) was reported in 2007 and the minimum (13) in 2020 (Figure 1).

**Figure 2:**
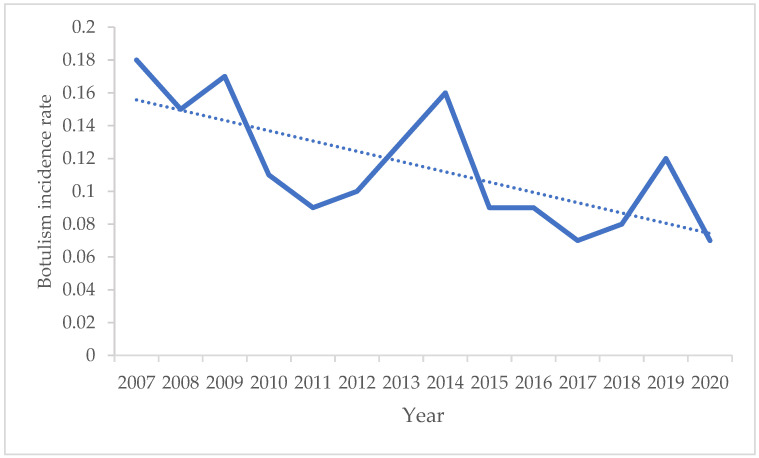
Annual incidence rate of botulism (number of botulism cases/100,000 inhabitants), Romania, 2007–2020.

The incidence rates declined over time in the study period, with the maximum incidence rate being recorded in 2007 as represented in Figure 2.

**Figure 3:**
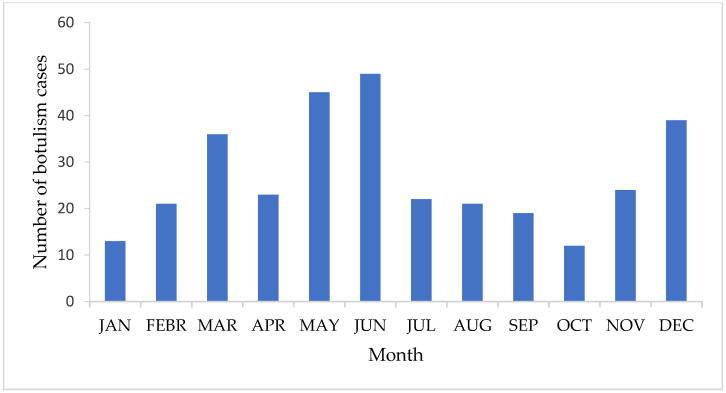
Monthly distribution of cumulative botulism cases (*n* = 325) in Romania, 2007–2020.

The cases were reported in every month of the year, with most of the cases in June (49, 15.1%), May (45, 13.8%) and December (39, 12.0%). The lowest number of cases was observed in October (12, 3.7%) as represented graphically in Figure 3.

### 3.2. Demographic Characteristics of Botulism Cases

**Figure 4:**
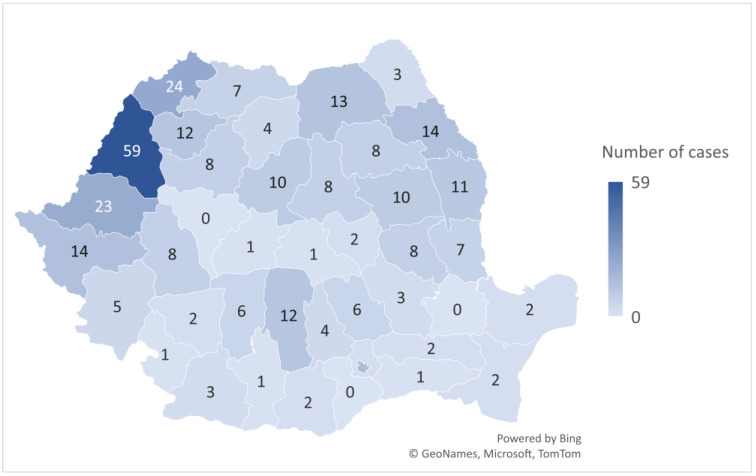
Distribution of cumulative botulism cases (*n* = 325) by county in Romania, 2007–2020.

Regarding geographical distribution, the largest number of cases were reported in the north and north-western parts of Romania, with Bihor County having the highest number of cases (59, 18.2%). In the southern and south-eastern counties, a small number of cases were reported (Figure 4). Three counties (Alba, Brăila, and Giurgiu), did not report botulism cases in the 2007–2020 period.

The disease was more common in rural areas compared with the urban ones (205, 63.1%). Also, men were more frequently affected than women (213, 63.5%) (Table 1).

**Figure 5:**
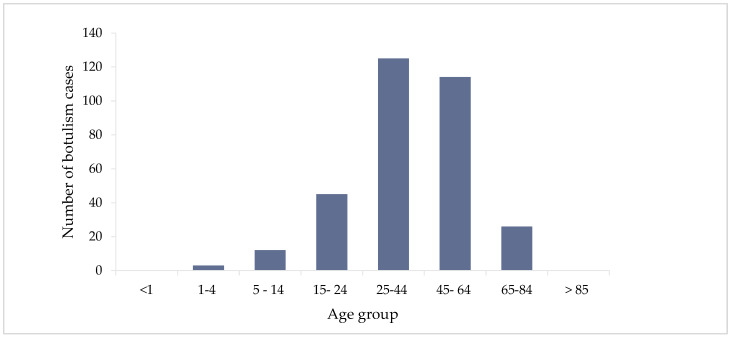
Distribution of cumulative botulism cases (*n* = 325) by age groups in Romania, 2007–2020.

Most cases were in the 25 to 44 years age group (125, 38.5%), followed by the 45–64 years age group (114, 35.1%). No cases were recorded in children under 1 year and in people over 85 years old (Figure 5).

### 3.3. Serological Diagnosis

Complete serological diagnosis, consisting of the identification of the botulinum toxin in patient serum using mice inoculation and the identification of the type of toxin using serum-neutralization was performed for 231 (79.9%) of the 289 confirmed cases. In almost all cases with serological diagnosis, the identified toxin type was BoNT/B (230, 99.5%). BoNT/E was recorded in a single case in 2007.

### 3.4. Epidemiological Investigation

Information on incriminated food items identified through epidemiological investigations was available for 161 cases, mostly being caused by food items prepared in the household (145, 90%). Sixteen (10%) cases reported commercial food products as the source of poisoning.

**Figure 6:**
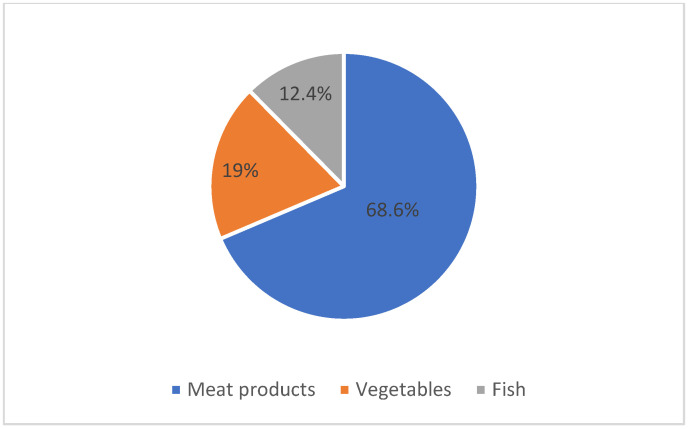
Foodborne botulism by food products linked to cases (*n* = 137), Romania, 2007–2020.

Data regarding the food categories involved were available for 137 cases. The main food category that led to the disease was represented by meat and meat-based products (94, 68.6%), followed by vegetables and fish (Figure 6).

### 3.5. Botulism Fatality

Between 2007 and 2020, there were 15 deaths recorded as caused by botulism in Romania, with a maximum of 3 deaths in 2009. The case fatality rate (CFR) was 4.6%. The number of deaths was higher among men (12), with a male/female ratio of 4/1.

Most deaths (11) were recorded among people from rural areas. The most affected age group was 65–84 years (7). The case fatality rate in this age group was 26.9% (Table 2).

## 4. Discussion

From the descriptive analysis of the data reported for 2007–2020, it was observed that botulism cases in Romania occurred every year, with a median of 22 cases per year. Both isolated cases and outbreaks have been reported [26]. The maximum number of cases was reported in 2007, when Romania ranked second in Europe in terms of number of reported cases, after Poland [27]. Additionally, a large number of cases were reported in 2009 (*n* = 29) and 2019 (*n* = 23), with Romania ranking first among EU/EEA countries [24,25,28,29]. According to the latest ECDC Surveillance Report, Romania, together with France, Italy, and Poland, is among the first four countries that reported the highest annual number of botulism cases in the last five years at the European level [2].

The average incidence value for the studied period was 0.1 per 100,000 population. In recent decades, in Romania, the incidence rate of botulism cases had a fluctuating evolution. After 1990, there was an increase in the incidence rate, from 0.06 ± 0.03 per 100,000 inhabitants in the period 1980–1989 to 0.1 ± 0.04 per 100,000 in the period 1990–1999, reaching the maximum value of 0.12 ± 0.04 per 100,000 in the decade 2000–2009 [30]. At present, according to national reports, the multi-year trend is still downward [24]. Factors influencing the decreasing multi-year trend were the improvements in surveillance methodology, involving the veterinary authorities and the sanitary education campaigns.

The analysis using the place of residence showed that more than half of the patients (205, 63.1%) acquired the intoxication in the rural environment, probably due to specific risk factors (poor hygienic and sanitary status compared to the urban environment, eating habits, etc.). A similar situation was described in Italy. The results of a study presenting the evolution of botulism for a period of 30 years (1986–2015) showed that most cases of foodborne botulism occurred in people from rural areas [1]. On the other hand, there are countries (Poland) where the incidence of botulism in rural areas decreased to the level of that is recorded in urban areas [31].

Regarding the distribution of cases by age group, in Romania, we encountered some peculiarities when compared to the European situation. In our study, approximately half of the cases (125, 38.5%) were reported in young adults (age group 25–44 years). During the analyzed period, no cases of botulism were registered in the age group under one year, although at the European level, this age category is the most affected, according to the ECDC reports [2]. Cases of infant botulism are reported all over the world, considering that this is the most frequently encountered clinical form, representing 70% of all new cases of botulism annually [32]. Another peculiarity observed for Romania was the fact that during the 14 years included in our analysis, no cases were reported in people over 85 years old, although studies prove that foodborne botulism also affects the elderly [33].

Regarding the clinical forms of botulism, all reported cases were represented by foodborne botulism. Unlike in the EU/EEA and in the United States of America (USA), no cases of wound botulism have been reported in Romania. Also, no case of botulism has been reported in drug users, although drug use has been increasing in recent years in Romania. According to the latest report on drugs in Romania (2022), increases were found for all types of consumption. A total of 10.7% of people aged between 15 and 64 years have consumed at least one type of illicit drug during their lifetime. For example, in Bucharest, the ratio of injection drug users is estimated at 3.3 (95% confidence interval: 2.1–7.3) persons/1000 inhabitants [34]. People who use injection drugs are at risk of wound botulism, therefore is important to increase awareness in hospitals and other healthcare facilities to support diagnosis and treatment as well as reporting to appropriate public health authorities.

Regarding the distribution of botulism cases at the county level, we found that most cases were reported in the north and north-west of the country, with a maximum of 59 cases in Bihor County (80, 18.2%). In contrast, there were also counties in which, during the 14 years included in the study, no cases of botulism were registered (Alba, Brăila, and Giurgiu). A possible cause for the large number of cases registered in the north-western part of the country could be the eating habits of the population in this region. In Transylvania, during the Christmas season, there are specific canned cuisine techniques such as the salting, fumigation, marinating, and maturation of pork and its organs for making meat-based products (“sausages”, “blood pudding”, “smoked bacon”) [35]. Moreover, studies have shown that most cases of botulism in north-west Romania occur in the winter–spring season, which further suggests that eating habits in the respective area, especially during the winter holiday season, contribute to an increased rate of occurrence [36]. Although most cases were recorded in May and June, there was no overall seasonality observed in our study. Even at the European level, the seasonality of the disease was not described; rather, random peaks were observed [2].

Regarding the food sources of botulism and their site of origin, the incriminated food item could not be identified in all cases involved in the epidemiological investigation or the information was difficult to reconstruct based on the existing data sources. From the analysis of the available data, we found that most of the foods that contributed to the onset of the disease were prepared at home (145/161, 90%). The main category of food products was represented by meat products (94/137, 68.8%). According to the ECDC reports, the same food category has been linked to the occurrence of botulism in France and Poland, which is in contrast to Italy where canned vegetables prepared at home are the main food products that have contributed to the transmission of the disease [2].

BoNT/B was identified in 99.5% of the cases in which complete serological diagnosis was performed, and only one case was identified as BoNT/E. These results are consistent with the types of food sources identified in our study, as it is known that BoNT/B is associated mainly with the consumption of pork products and BoNT/E with the consumption of fish products [17,37]. This result is similar to that reported in Europe, with type B of the toxin being identified in most cases of botulism. In the period 2017–2021, BoNT/B determined 89% of human botulism cases in the EU/EEA [2]. Type E of the toxin is less often identified, but outbreaks have also been reported, including in Europe. For example, in 2016, a cross-border outbreak was reported with five cases in Germany and Spain, which occurred as a result of the consumption of dried fish products [38].

In our study, 15 deaths were recorded during the analyzed period, with a male/female ratio of 4/1, and a significant difference was also noted in terms of residence environment; the majority (12) were registered in rural areas. This difference may be due to the low accessibility to medical services, which are more difficult to access in comparison with the ones in urban areas. This is in addition to the population’s lack of knowledge about the risk of botulism and non-compliance with disease prevention measures.

The case fatality ratio of botulism in Romania for the interval of 2007–2020 was 4.6%, which is within the lower end of the range mentioned in the literature, between 3 and 10% [17]. This could reflect the early recognition of cases and the rapid administration of botulinum antitoxin, even if the diagnosis of botulism, especially in sporadic cases, could be a challenge for the clinicians [32].

A limitation of the study is the fact that it was a retrospective study, based on the data available in the surveillance forms, annual reports, and epidemiological investigations reports. In some cases, the data were incomplete, and difficult to reconstruct, especially regarding food items linked to foodborne botulism. In addition, the available data did not allow us to identify and describe all the botulism outbreaks that have occurred in Romania during the entire study period.

Educating the population by raising awareness of the risk of disease occurrence, compliance with food safety measures, and presenting oneself at medical centers at the slightest suspicion of disease for the early administration of botulinum antitoxin is essential. By increasing awareness that the disease is severely aggressive and has a high mortality rate, the burden on the medical system can be avoided. In addition, increasing efforts are needed to raise awareness among all healthcare providers to avoid misdiagnosis, especially due to the low prevalence of the disease and similarity with other diseases in terms of symptoms. The importance of rapid notification of cases to public health authorities should be reinforced in order to start the epidemiological investigation as soon as possible. The rapid identification of the incriminated food item and its removal from consumption could prevent additional cases. Future research directions may also be highlighted.

There is a dire need to raise awareness of the risks of wound botulism in intravenous drug users, using measures to inform the general population, as well as the medical staff, considering the recent increase in the number of cases in this risk category, even in developed countries.

## 5. Conclusions

Botulism is a priority disease for the surveillance system in Romania, with foodborne cases reported every year, especially in rural areas in the north and north-west counties. The incidence of botulism had a multi-year downward trend during the study period due to improvements in the surveillance methodology and due to the involvement of the veterinary authorities. The highest fatality rate was observed among seniors. Household-prepared food was the source for most cases and the BoNT/B toxin was the type predominantly found. Complete and timeline surveillance data are essential for the implementation of control measures and for adapting educational campaigns according to existing needs.

## Figures and Tables

**Table 1:** Demographic characteristics of cumulative botulism cases (*n* = 325) in Romania, 2007–2020.

Characteristics		Total Cases (*n* = 325)
Residence	Urban	120 (36.9%)
	Rural	205 (63.1%)
Gender	Female	112 (34.5%)
	Male	213 (65.5%)

**Table 2:** Number of deaths, number of cases and case fatality rate from botulism by age group (*n* = 325) in Romania, 2007–2020.

Age Group	Number of Deaths	Number of Cases	Case Fatality Ratio (%)
under 1 year	0	0	0
1–4 years	0	3	0
5–14 years	0	12	0
15–24 years	0	45	0
25–44 years	2	125	1.6
45–64 years	6	114	5.3
65–84 years	7	26	26.9
Over 85 years	0	0	0
**Total**	**15**	**325**	**4.6**

## Data Availability

The data analyzed during the current study are available on the National Institute of Public Health website (https://insp.gov.ro/centrul-national-de-supraveghere-si-control-al-bolilor-transmisibile-cnscbt/rapoarte-anuale/, last accessed: 27 March 2024) and from the corresponding author upon reasonable request.
